# CD8^+^ T-cell pathogenicity in Rasmussen encephalitis elucidated by large-scale T-cell receptor sequencing

**DOI:** 10.1038/ncomms11153

**Published:** 2016-04-04

**Authors:** Tilman Schneider-Hohendorf, Hema Mohan, Christian G. Bien, Johanna Breuer, Albert Becker, Dennis Görlich, Tanja Kuhlmann, Guido Widman, Sebastian Herich, Christiane Elpers, Nico Melzer, Klaus Dornmair, Gerhard Kurlemann, Heinz Wiendl, Nicholas Schwab

**Affiliations:** 1 Department of Neurology, University of Münster, 48149 Münster, Germany; 2 Epilepsy Center Bethel, Krankenhaus Mara, 33617 Bielefeld, Germany; 3 Institute of Neuropathology, University Hospital Bonn, 53105 Bonn, Germany; 4 Institute of Biostatistics and Clinical Research, University of Münster, 48149 Münster, Germany; 5 Department of Neuropathology, University of Münster, 48149 Münster, Germany; 6 Department of Epileptology, University of Bonn, 53127 Bonn, Germany; 7 Children's Hospital of the University Medical Center, University of Münster, 48149 Münster, Germany; 8 Institute for Clinical Neuroimmunology, Ludwig-Maximilians-University Munich, 80539 Munich, Germany; 9 Biomedical Center, Ludwig-Maximilians-University Munich, 80539 Munich, Germany; 10 Munich Cluster for Systems Neurology (SyNergy), Ludwig-Maximilians-University Munich, 80539 Munich, Germany

## Abstract

Rasmussen encephalitis (RE) is a rare paediatric epilepsy with uni-hemispheric inflammation and progressive neurological deficits. To elucidate RE immunopathology, we applied T-cell receptor (TCR) sequencing to blood (*n*=23), cerebrospinal fluid (*n*=2) and brain biopsies (*n*=5) of RE patients, and paediatric controls. RE patients present with peripheral CD8^+^ T-cell expansion and its strength correlates with disease severity. In addition, RE is the only paediatric epilepsy with prominent T-cell expansions in the CNS. Consistently, common clones are shared between RE patients, who also share MHC-I alleles. Public RE clones share Vβ genes and length of the CDR3. Rituximab/natalizumab/basiliximab treatment does not change TCR diversity, stem cell transplantation replaces the TCR repertoire with minimal overlap between donor and recipient, as observed in individual cases. Our study supports the hypothesis of an antigen-specific attack of peripherally expanded CD8^+^ lymphocytes against CNS structures in RE, which might be ameliorated by restricting access to the CNS.

## Abstract (web summary)

Rasmussen Encephalitis is a rare neurological disease accompanied by inflammation and T cell infiltration in the brain. Here the authors show that the severity of this disease correlates with clonal CD8 T cell expansion.

## Graphical abstract

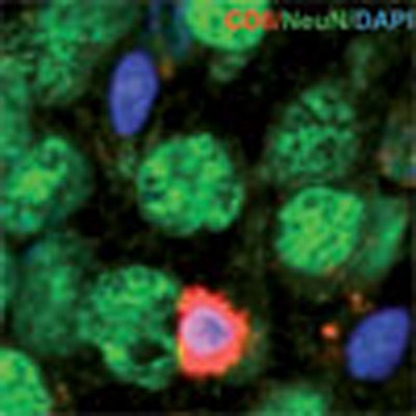

Rasmussen encephalitis (RE) is a rare chronic brain disorder characterized by uni-hemispheric inflammation, intractable seizures and progressive neurological deficits^1^,^2^,^3^,^4^. The disease mainly affects children with an average age of onset of 6–7 years^5^,^6^. However, RE has also been reported in adolescent and adult patients accounting for 10% of all cases^7^,^8^. Histopathological analysis of the brain specimens demonstrates inflammation with microglial nodules, perivascular and parenchymal infiltration of T cells, neuronal and astrocytic loss and gliosis of the affected hemisphere^9^,^10^. The hypothesis of RE being a primarily antibody-driven disease (for example, antibodies against subunit 3 of the α-amino-3-hydroxy-5-methyl-4-isoxazolepropionic acid receptor (GluA3))^11^ was shown to be disease unspecific^12^,^13^. Involvement of T cells in the pathogenesis of RE is being considered more relevant. Farrell *et al*.^9^ showed that most of the inflammatory cells in the RE brain specimens were T lymphocytes along with activated microglial cells. Another study revealed that most of the infiltrating T lymphocytes were CD8^+^, 7% of them were found in close apposition to neurons and contained granzyme B granules directed towards the major histocompatibility complex class I positive neurons^3^. Granzyme B^+^ CD8^+^ cells were not only directed against neurons but also against astrocytes in RE brain specimens bringing about astrocytic degeneration along with neuronal cell death^14^,^15^.

The precise antigen triggering the T-cell response is currently not known. However, there are a few studies analysing the T-cell receptor (TCR) repertoire in the brain and peripheral blood of RE patients^15^,^16^. Our previous work using TCR spectratyping showed for the first time that CD8^+^ T-cell clones found in the brain are expanded in the periphery as well and can persist for years^15^.

Using a large collection of biospecimens from RE patients (*n*=23) from different compartments (brain, cerebrospinal fluid (CSF) and blood), we here employed a new method of high-throughput sequencing coupled with multiplex PCR^17^,^18^, which allows determination of the TCR repertoire complexity of RE patients in depth. This methodology allowed us to determine copy number and nucleotide sequence of a specific clone at the same time. As study controls we included peripheral blood (and CNS specimen) from paediatric patients suffering from temporal lobe epilepsy, cortical dysplasia, common colds or headaches (the latter two without any CNS pathology) to elucidate whether and how the immune response in periphery and CNS differs from RE. Our analysis reveals that RE is characterized by disease-specific clonal expansions in the CNS and we provide sequence evidence that clonal expansions are a prominent feature of RE, thereby showing that antigen-specific clonal responses occur and persist in the brain and peripheral blood of RE patients. The degree of these peripheral expansions correlates with disease severity, and the public clones shared between RE patients have common biochemical characteristics. In addition, we provide data on RE patients treated with one monoclonal antibody (basiliximab), two separate monoclonal antibodies (rituximab and natalizumab) or with two rounds of stem cell transplantation (autologous and syngeneic), providing insights into T-cell plasticity during treatment, but also into RE pathogenesis, as well as possible treatment options for RE patients in the future.

## Results

### RE blood CD8^+^ TCR perturbation corresponds to brain atrophy

The clonal diversity of the peripheral blood CD8^+^ TCR repertoire from 23 RE patients including 6 adult-onset cases and age- and sex-matched children suffering from temporal lobe epilepsy (TLE; *n*=4), cortical dysplasia (CD; *n*=4), headache (*n*=8) and common cold (*n*=5) were assessed by high-throughput sequencing. TCR entropy is a statistical measure for the degree of perturbation. High entropy demonstrates more normally distributed TCR repertoires, while low entropy is found in samples with strong clonal expansions (Methods). Clonal expansions consist of T-cell clones, which are defined by an identical TCR nucleotide sequence and identified by sequencing the hypervariable and antigen-binding complementary determining region 3 (CDR3) including variable (V), joining (J) and diversity (D) region(s) of the TCR variable β chain.

**Figure 1:**
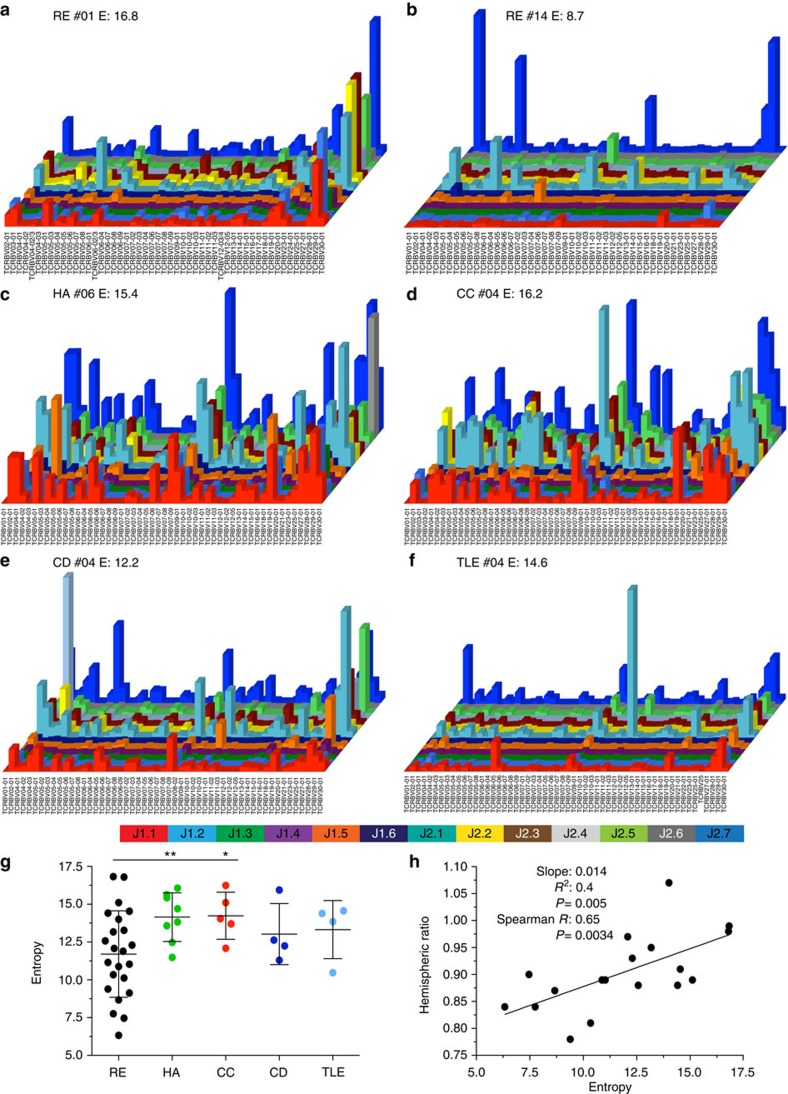
RE blood CD8^+^ TCR entropy correlates with disease severity. Shown are the peripheral CD8^+^ TCR repertoires of representative patients for (**a**) RE with high entropy (E), (**b**) RE with low entropy, (**c**) headache (HA), (**d**) common cold (CC), (**e**) CD and (**f**) TLE. The *x* axis lists all analysed Vβ genes, the *z* axis the Jβ genes and the column height indicates the total reads of this specific V/J combination; (**g**) a quantification of the repertoire diversity by showing the CD8^+^ repertoire entropy of each patient group (RE: black, *n*=23; HA: green, *n*=8; CC: red, *n*=5; CD: dark blue, *n*=4; TLE: light blue, *n*=4, mean±s.d., unpaired *t*-test with Welch's correction). (**h**) Correlation of the RE cohort (*n*=18) with respect to their entropy (*x* axis) and disease severity (hemispheric ratio; *y* axis) (non-parametric Spearman correlation with Gaussian approximation).

Figure 1a–f shows examples of the peripheral blood TCR repertoire entropy of the patient groups with one example of an RE patient with high entropy (Fig. 1a), low entropy (Fig. 1b) and representative examples for headache, common cold, CD and TLE (Fig. 1c–f). RE patients' peripheral blood CD8^+^ TCR repertoire entropy was lower as compared with the non-CNS disease control groups headache (*P*=0.007) and common cold (*P*=0.020), while the differences to the childhood epilepsy control groups, TLE and CD did not reach statistical significance (Fig. 1g, unpaired *t*-test with Welch's correction). The entropy of the TCR repertoire did not differ between early-onset and adult-onset RE patients (Supplementary Fig. 1A), a correlation between their age and clonal perturbation in terms of entropy could also not be detected (Supplementary Fig. 1B), allowing us to analyse these patients as one group. In all, 7 of 23 RE patients (30.4%) presented with monoclonal expansions (one peripheral blood T-cell clone comprising more than 5% of the productive TCR repertoire), which in the individual patients consequently lead to severe shifts in the respective Vβ and Jβ usage, as well as CDR3 length distribution, but on average there was no significant Vβ/Jβ gene preference in the RE cohort when compared with controls (Supplementary Fig. 1C–E). Consistent with the hypothesis of RE being a CD8^+^ T cell-mediated CNS disorder, a strong correlation could be found, when comparing the RE patients' CD8^+^ TCR entropy to the clinical parameter hemispheric ratio, which is a magnetic resonance imaging measurement of uni-hemispheric atrophy in RE^10^. Patients with a greater degree of peripheral blood CD8^+^ TCR perturbation (=lower entropy) also presented with increased hemispheric atrophy (=lower hemispheric ratio; Fig. 1h, Spearman *R*=0.65; *P*=0.0034, non-parametric Spearman correlation, Gaussian approximation).

### Clonal T-cell expansions are specifically present in RE CNS

**Figure 2:**
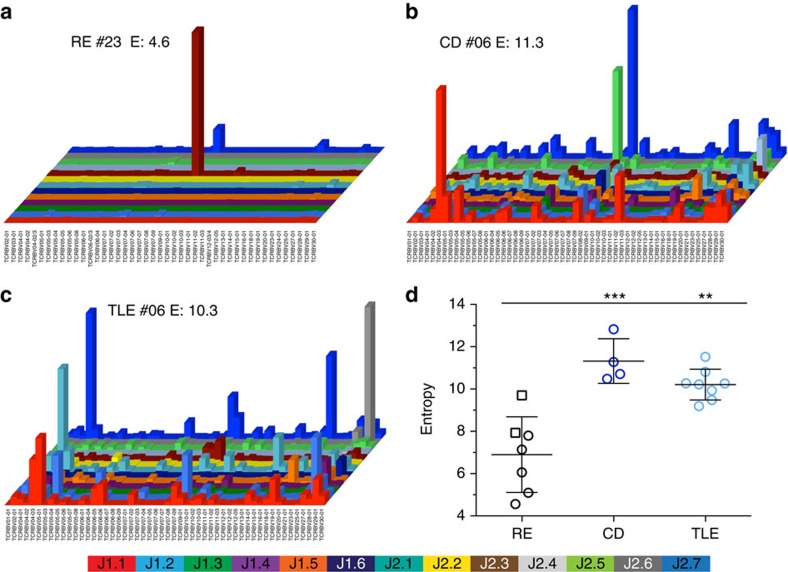
RE patients' CNS TCR repertoire is highly restricted. Shown are the CNS TCR repertoires of representative patients for (**a**) RE, (**b**) CD, (**c**) TLE. The *x* axis lists all analysed Vβ genes, the *z* axis the Jβ genes and the column height indicates the total reads of this specific V/J combination; entropy is given. (**d**) Quantification of the repertoire diversity by showing the TCR repertoire entropy of each patient group (RE: black *n*=7; CD: dark blue, *n*=4; TLE: light blue, *n*=8, mean±s.d., unpaired *t*-test with Welch's correction). Open circles indicate brain biopsy specimen and open squares indicate CSF samples.

As expected, clonal perturbations were in general more prominent in CNS samples of patients with paediatric epilepsies compared with the peripheral TCR repertoire, resulting in lower entropy overall (one representative example shown in Fig. 2a–c, respectively). Five of five RE CNS samples (100%) showed pronounced monoclonal expansions (one central T-cell clone comprising more than 10% of the productive T-cell repertoire), whereas none of the two RE CSF samples and only one out of four CD (25%) and one out of eight TLE CNS samples (12.5%) showed a monoclonal expansion. The entropy of RE CNS samples including CSF (mean 6.9±s.d. 1.8) was clearly lower compared with the paediatric epilepsy controls CD (mean 11.3±s.d. 1.1; *P*=0.0009) and TLE (mean 10.2±s.d. 0.7; *P*=0.0026) indicating a pronounced clonally restricted, antigen-driven immune reaction in the CNS of RE patients (Fig. 2d, unpaired *t*-test with Welch's correction). Similar to the peripheral blood samples, there were only individual Vβ/Jβ preferences in the CNS of RE patients due to the strong monoclonal expansions, which on average did not result in a disease-specific Vβ/Jβ usage in the CNS, when compared with controls (Supplementary Fig. 2A–C). Interestingly, the immense monoclonal expansions from RE patient #01, #02 and #23 were comprised of numerous silent nucleotide substitutions (291, 11 and 18, respectively) and accompanied by similar T-cell clones of the same Vβ/Jβ combination and CDR3 length with only one amino-acid substitution (21, 1 and 22, respectively), which could not be found in the TLE (20 and 5, respectively) and CD (5 and 2, respectively) monoclonal expansion to this extent (Supplementary Table 2).

### RE CNS T-cell expansions are shared with the CD8 periphery

**Figure 3:**
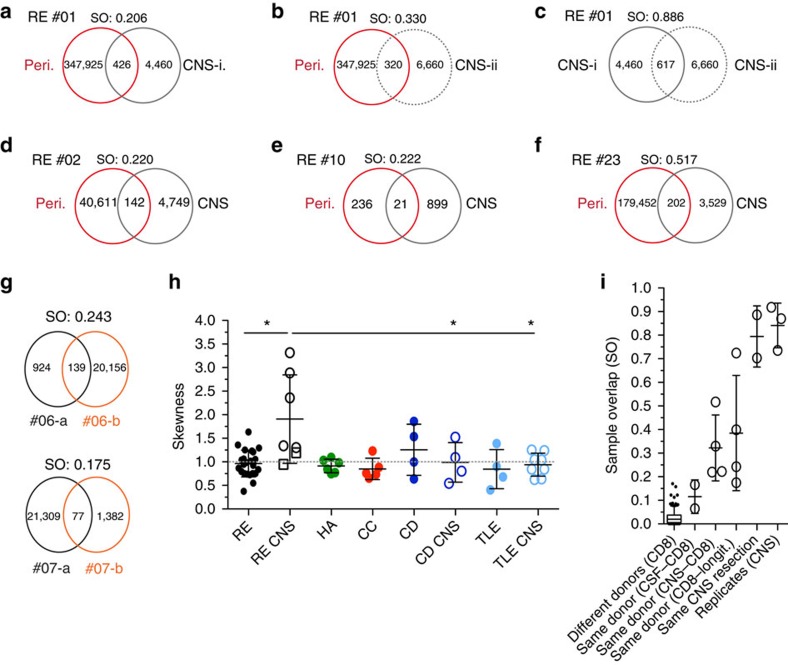
Expanded CD8^+^ T-cell clones shared between periphery and CNS. Shown are four RE patients with corresponding periphery and CNS samples. From patient RE #01 two CNS tissue resections were available. The Venn diagrams show the productive uniques of each sample, their shared productive uniques (shared clonotypes/CD8^+^ T-cell clones), the SO and the top 10 shared clones of the respective sample comparison (given are amino-acid sequence, Vβ gene, Jβ gene and frequencies in the respective sample; clones are sorted by frequency in the CNS sample). (**a**) RE 01 CD8^+^ periphery and CNS-i (first tissue resection), (**b**) RE 01 CD8^+^ periphery and CNS-ii (second tissue resection), (**c**) RE 01 CNS-i and CNS-ii, (**d**) RE 02, (**e**) RE 10 and (**f**) RE 23. (**g**) Two RE patients where peripheral CD8^+^ T-cell samples were available 1 year apart (a and b), (**h**) the skewness of the different patient groups and compartments described in Figs 1 and 2 (RE per. black, *n*=23; RE CNS black, *n*=7; HA green, *n*=6; CC green, *n*=5; CD dark blue, *n*=4; CD CNS dark blue, *n*=4; TLE periphery (per). light blue, *n*=4; TLE CNS light blue, *n*=8). Closed circles indicate peripheral CD8^+^ T-cell samples, open circles indicate CNS tissue specimen and open squares indicate CSF samples (mean±s.d., unpaired *t*-test with Welch's correction). (**i**) Comparison of SOs between CD8^+^ compartments of different donors (*n*=231, longitudinally (longit.) whiskers: 5–95 percentile), CD8^+^ and CSF of the same donor (*n*=2), CD8 and CNS of the same donor (*n*=4), CD8^+^ from two time points and the same donor (*n*=4), two tissue resections of the same donor's brain tissue (*n*=2), and the same CNS sample sequenced twice independently (*n*=3) (mean±s.d.).

We analysed four patients with corresponding CNS^+^ and blood samples (patient #01, #02, #10 and #23) for similarities between the two compartments and could detect the top 10 T-cell clones, which are expanded in the CNS, also in the periphery (Table 1). For patient #01 two CNS biopsies from different regions of the brain were available. Sample overlap (SO) is computed by averaging across the two ratios of shared reads over total reads for each sample. Whereas only 10–15% of all TCRs were found in both regions due to the limited sequencing depth, 7 of the top 10 CNS-expanded clones matched between the two regions resulting in a sample overlap of 0.886, which is in the range of technical replicates (Table 1 and Fig. 3a–c,i). The top shared clone from patient #01 with the CDR3 amino-acid sequence CASSGYEQYF constituted 49% of the T-cell infiltrate of region i and 45% of region ii. Interestingly, in both regions a T-cell clone with an amino-acid substitution (D for E, resulting in a different *Jβ* gene 1–2) was among the top 10 resulting in a CDR3 sequence CASSGYDYTF (Table 1). Despite the fact that peripheral blood comprises far more T cells (and T-cell clones), the sample overlap between the periphery and both CNS regions was comparably high (Fig. 3i). Similarly, in the CNS of patient #02 a single monoclonal expansion of Vβ11-2–Jβ2-3 accounted for 27% of the TCR repertoire. This specific TCR was also present, if not strongly expanded, in peripheral blood (Table 1 and Fig. 3d). In patient #10, who suffered from slowly progressing adult-onset RE, the highest monoclonal expansion of the CNS (12.2%, CASSYWRGRIFDEQFF) was not shared with the peripheral repertoire. Other expanded and shared clones were observed as well, albeit with a lower frequency in the CNS and a higher frequency in the periphery (Table 1 and Fig. 3e). Patient #23 showed the strongest monoclonal expansion of the overall 73 study samples in his CNS with 53% (Vβ7-9–Jβ2-3; CASRTTGPNDTQYF) with a correspondingly high frequency of 0.6% in peripheral blood (Table 1 and Fig. 3f). There were two patients in our cohort (#06 and #07), where we had access to peripheral blood from two time points (1 year apart). Comparing their TCR repertoire revealed that the expanded T-cell clones persisted in the periphery. Patient #06 and #07 had a SO of 0.243 and 0.175, respectively (Fig. 3g,i). *R*^2^ was 0.951 and 0.402, respectively, where the correlation of frequencies for all shared clones is given by *R*^2^.

Since the CDR3 length of the top shared CNS clones was often unusually short (below 15 amino acids or 45 nucleotides) we analysed the CDR3 length distribution for all cohorts and both compartments for skewness^19^ (Supplementary Fig. 3). RE CNS samples (including the two CSF samples) on average showed a deviation from the normal, or Gaussian, distribution, which was skewed towards shorter fragment lengths when compared with the peripheral RE samples (*P*=0.039), CD CNS samples (*P*=0.049) and TLE CNS samples (*P*=0.038) (Fig. 3h,i, unpaired *t*-test with Welch's correction).

### Public RE clones share CDR3 length and Vβ usage

We analysed the top 100 expanded T-cell clones of the 23 RE patients and 23 paediatric controls for their potential presence (defined by the same CDR3 amino-acid sequence including the same Vβ and Jβ combination) in the complete peripheral blood CD8^+^ sequencing data of these 46 patients). This comparison resulted in 330 clones, which were exclusively found in at least two RE patients (and none of the controls, =‘RE-specific'), 256 clones, which were exclusively found in at least two controls (and none of the RE patients, =‘Ctrl-specific', Supplementary Table 3), and 4,498 clones, which were found in either one RE patient, one Ctrl patient or one of both patient groups (=‘unspecific'). The most frequent RE-specific public clone was found in 52% of RE patients, followed by the monoclonal CNS expansion of patient #01, which was exclusively found in 39% of RE patients including two CNS samples. One clone was found in three of five CNS samples/corresponding blood, as well as one additional RE patient. The most common clone was present in 65% of all control samples (*n*=23) and 57% of all RE patients (*n*=23) (Table 2).

**Figure 4:**
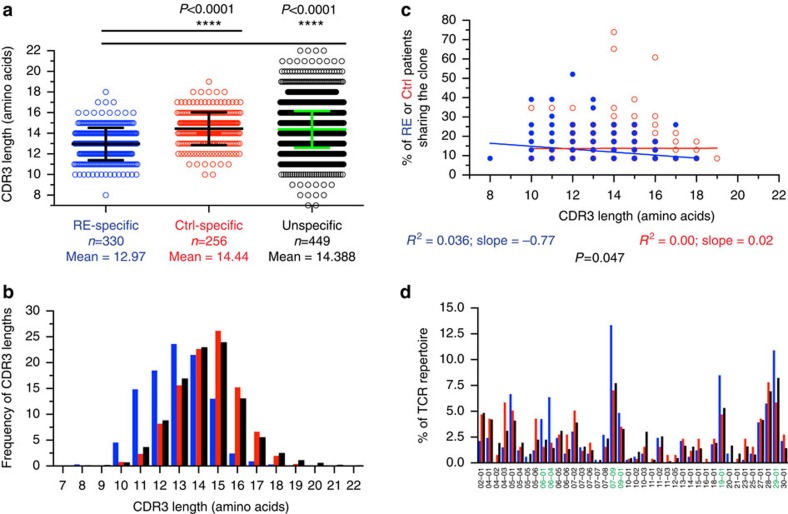
RE-specific clones share shorter CDR3 regions and Vβ genes. (**a**) Shown is the mean CDR3 length (in amino acids) of the previously defined groups of clones (RE-specific, *n*=330; Ctrl-specific, *n*=256 (only found in control patients), and unspecific, *n*=4,498 (found, but not shared between either one RE patient, one control patient or in one of each)) (mean±s.d., unpaired *t*-test with Welch's correction). (**b**) CDR3 length distribution of RE-specific (blue), Ctrl-specific (red) and unspecific (black) clones. (**c**) Correlation between the CDR3 length (in amino acids) of the previously described groups of clones and the percentage of RE patients (blue) or controls (red), who shared this clone. Lines indicate the linear regression (analysis of covariance). (**d**) Vβ genes of the previously described groups of clones. Over-expressed Vβ genes in RE-specific clones are highlighted in green. Ctrl, control.

In line with the finding of a skewed repertoire in RE CNS samples, we could find that only RE-specific clones had significantly shorter CDR3 lengths (mean amino-acid length 12.97), whereas the fragment lengths of Ctrl-specific and unspecific public clones presented with the known average mean of the Gaussian distribution of CDR3 fragment lengths (mean amino-acid length 14.44; *P*<0.0001; and 14.38; *P*<0.0001, respectively, unpaired *t*-test with Welch's correction) (Fig. 4a), resulting in a shifted CDR3 fragment length distribution exclusively in RE-specific clones (Fig. 4b). Consistently, the likelihood for a clone with decreasing CDR3 length to be shared between patients was only increasing in RE patients, whereas no correlation could be found in controls (Fig. 4c, *P*=0.047, linear regression and analysis of covariance). In addition, Vβ gene distribution among RE-specific public clones was perturbed, due to over-represented Vβ genes *Vβ06-01*, *Vβ06-04*, *Vβ07-09*, *Vβ09-01*, *Vβ19-01* and *Vβ29-01* comprising 48% of all used Vβ genes in RE-specific public clones, as compared with 25% in Ctrl-specific clones and 28% in unspecific clones (Fig. 4d). These ‘RE-specific' Vβ genes could also be found in 15 of 25 (60%) CNS-resident, RE-specific common clones. In the context of public clones, it is important to mention that 17 of 22 RE patients (77%) share the HLA-C 07 phenotype (allelic frequency: 22/44=50%) (Table 3).

### TCR repertoire changes after stem cell transplantation

**Figure 5:**
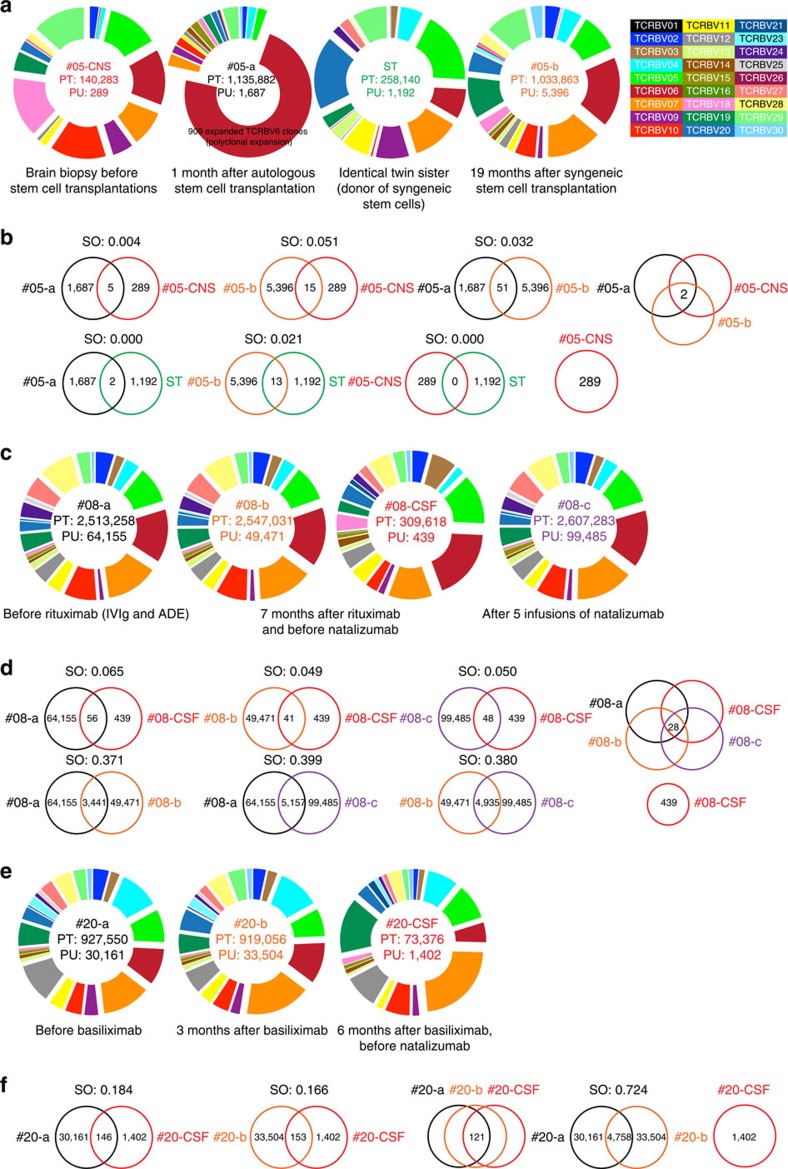
Experimental therapies for RE. (**a**) Shown are the Vβ family distribution in a CNS sample before the stem cell transplantations (red), in a peripheral CD8^+^ T-cell sample 1 month after the autologous stem cell transplantation (black), in the syngenic twin sister (ST; green) and in a peripheral CD8^+^ T-cell sample 19 months after the syngeneic stem cell transplantation (orange). Given are PT and PU. (**b**) Given are the SOs between the different samples and the productive uniques, as well as shared clonotypes (in the Venn diagrams). Listed are the shared clonotypes (amino-acid sequence, Vβ and Jβ genes, frequency in either peripheral samples (peri.) or the CNS. (**c**) Shown are the Vβ family distribution in a peripheral CD8^+^ T-cell sample before rituximab (black), 7 months after rituximab treatment and before treatment with natalizumab (orange), after 5 infusions of natalizumab (purple), and in a CSF sample after treatment with rituximab and before treatment with natalizumab (red). Given are PT and PU, as well as the top 10 CDR3 sequences of the CSF sample. (**d**) Given are the SO between the different samples and the productive uniques, as well as shared clonotypes (in the Venn diagrams). Listed are the shared clonotypes (amino-acid sequence, Vβ and Jβ genes, frequency in either peripheral samples (peri.) or the CSF. (**e**) Shown are the Vβ family distribution in a peripheral CD8^+^ T-cell sample before basiliximab (black), 3 months after basiliximab treatment and before treatment with natalizumab (orange), and in a CSF sample 6 months after treatment with basiliximab and before treatment with natalizumab (red). Given are PT and PU. (**f**) Given are the SO between the different samples and the productive uniques, as well as shared clonotypes (in the Venn diagrams).

Patient #05 in this study with corresponding CNS and peripheral specimens (see Fig. 3 for the four other patients) underwent immune ablation and stem cell reconstitution with haematopoietic stem cells (CD34^+^) twice, once autologous and the second time from her syngeneic twin sister. We analysed corresponding CNS tissue and peripheral blood from two time points, the first 1 month after autologous, the second 19 months after syngeneic stem cell transplantation. The immune ablative regimen with subsequent autologous stem cell transplantation resulted in an immense over-representation of Vβ family 6 due to a polyclonal expansion comprising 909 different clones making up 62% of the TCR, which was not found in the corresponding CNS or any other assessed sample to this extent. In addition, the autologous stem cell transplantation resulted in a very low number of individual productive unique clones (PU), when compared with the total productive reads of the sample (PT). The patient's condition did not improve, which is why she was subjected to a second, syngeneic stem cell transplantation, after which her PU clones increased about fivefold with a comparable number of PT reads (Fig. 5a). The CNS biopsy was collected before the stem cell transplantations and should, therefore, contain putatively pathogenic T-cell clones. Comparing the Vβ repertoire for shared clones between the CNS and periphery after the stem cell transplantations, as expected, we found very few shared clones (5 and 15, respectively) at the first and second time points (Fig. 5b). Also the two longitudinal peripheral blood time points of patient #05 showed few similarities, together indicating the development of a new TCR Vβ repertoire after both ablative regimes. The Vβ repertoire of the syngeneic twin sister was completely different from that of the patient after the autologous stem cell transplantation (SO: 0.000) but also lower than expected after the syngeneic stem cell transplantation (SO 0.021) (Fig. 5b), again hinting towards a donor-independent development of the new repertoire. Interestingly, two clones, which were present in the CNS tissue, were expanded and persistent at both peripheral time points after immune reconstitution (Supplementary Table 4).

### TCR repertoire changes after rituximab and natalizumab

Our cohort also included two patients with corresponding CSF and peripheral specimens, who were undergoing experimental therapies. Patient #08 in our study was treated with the monoclonal antibody rituximab (1 cycle, 2 × 1,000mg) which depletes CD20^+^B cells. This treatment, however, did not alter the seizure frequency, which is why she was subsequently treated with another monoclonal antibody (natalizumab) 7 months later^20^. Natalizumab is an anti-CD49d antibody, which acts by inhibiting the migration of immune cells across the blood–brain barrier^21^. From this patient we analysed peripheral blood from three time points: before rituximab; 7 months after rituximab (also T0 time point for natalizumab); and 5 months after natalizumab treatment. Seven months after rituximab treatment the absolute number of unique clonotypes decreased by 23%. In contrast to this, after five infusions of natalizumab the absolute number of unique clonotypes increased by 102%. In contrast, the expression of individual Vβs or Jβs remained constant over time (Fig. 5c) and was comparable to the CSF (before treatment with natalizumab). When comparing the peripheral blood time points with the CSF, we found that 28 clones were expanded and persisted over time with the CSF having the highest frequency for the respective clonotype (Supplementary Table 4). Comparing the TCR Vβ repertoire between any two given time points we found an expected degree of common and persistent clonotypes, as well as sample overlap between them (SO: 0.371, 0.399 and 0.380) (Fig. 5d).

### TCR repertoire changes after basiliximab

The second patient with corresponding CSF–peripheral blood samples (RE patient #20) was experimentally treated off-label with basiliximab, a monoclonal antibody directed against the interleukin-2 receptor alpha chain CD25 (ref. 22). While this drug is usually reserved for patients with renal transplants, the blockade of CD25, which is expressed on activated T cells, was hypothesized to dampen the inflammatory immune response in this RE case. However, the patient's condition only improved slightly during 3 months of treatment with basiliximab. His frequency of nightly seizures was reduced, whereas his epilepsia partialis continua remained unchanged. No changes in the TCR Vβ repertoire of the two peripheral time points could be detected concerning Vβ family usage, PT and PU in line with an SO of 0.724 (Fig. 5e,f). After the patient suffered a herpes zoster reactivation event, the treatment with basiliximab was stopped. Interestingly, after the 3 months of basiliximab therapy, a T-cell clone could be detected in peripheral blood as the top expanded clone with 4.3%, which was not found in the first peripheral sample, but was also found in the CSF 3 months later (Supplementary Table 4). This clone might be connected to the cellular immune response against herpes zoster. Similar to patient #08, a comparably extremely low SO was found between both time points (0.184 and 0.166, respectively) and the subsequent CSF sample (also see Fig. 3i). This patient was later also started on natalizumab therapy.

## Discussion

Previous studies have suggested a pathogenic role of CD8^+^ T cells in RE. This study was designed to provide an extensive data set of TCR sequences using a new technology, which allows determination of clonal frequencies with parallel sequence identification. We here used a uniquely large collection of biospecimens from RE patients, which allowed longitudinal assessments, comparison of different compartments (peripheral blood, CSF and CNS) and monitoring the consequences of different immune therapies (monoclonal antibodies rituximab, natalizumab, basiliximab, as well as autologous and syngeneic haematopoietic stem cell transplantation). Our findings are evidence that clonal expansions of CD8^+^ T cells are a crucial pathogenetic element in RE. Data from this study show that putatively pathogenically relevant expansions of CD8^+^ T cells correlate with disease severity in RE and T-cell expansions are a pathological feature specific for RE in contrast to other paediatric epilepsies. Furthermore, RE-specific public clones exist in CNS and periphery and show perturbed TCR Vβ gene distribution and shorter CDR3 lengths.

The finding that RE patients' CD8^+^ T cells show strong clonal expansions in their CNS is in line with previous studies^15^,^16^. The clonal expansions in the periphery were less pronounced, but correlated with uni-hemispheric atrophy. Together, this extends the presumed assumption that clonally expanded CD8^+^ T cells are causally linked to the triggering and perpetuation of RE pathology. The finding that the TCR repertoire of the CNS showed a higher overlap with the periphery than the CSF with the periphery could mean that expanded T cells might not enter from the periphery via the blood–CSF barrier (or expand in the CSF space), as has been hypothesized in multiple sclerosis, but over the blood–CNS barrier^23^,^24^. Clonal expansions of the CSF did not show prominent overlap with their peripheral counterparts. This might be explained by local expansion of T cells in the CSF space, which has previously been shown for B cells producing the oligoclonal bands in CSF of multiple sclerosis patients^25^.

Until recently, public clones between patients were very rarely detected, as the techniques to detect clonal expansions relied on length fragment distribution in gels and capillaries^26^. Therefore, the probability to find two individuals showing the same expanded clone was reserved to strong antigens^27^ or to biochemically similar clones between patients^28^. However, with the technique used in our study, it has been shown that the overlap between any two individuals is up to 7,000 times higher than anticipated with examples in the range of 10,000–15,000 shared clonotypes in naive CD8^+^ T cells and up to 3,000 in the CD8^+^ memory compartment^29^. To evaluate if the public clones found in our study could have pathological significance, we applied a very strict algorithm: only clones shared in at least two RE patients (of 23), but not found in any control (of 23) could be considered ‘RE-specific'. We found that these clones in contrast to clones that were only found in control patients shared some characteristics with regards to their CDR3 structure: the CDR3 region was significantly shorter and six Vβ genes were by far over-represented in these clones. Interestingly, the public clones, which were also found in CNS samples, presented with even higher percentages of these Vβ genes (60%). These findings fit to the skewness of the CNS repertoire in RE patients, which also hinted at the over-expression of shorter CDR3 regions. One possible explanation could be that this is due to these clones stemming from earlier periods of development, as it has very recently been shown that the CDR3 length distribution in fetal blood is significantly shorter than in infants^30^ and further increases with age, due to antigen exposure^31^. This could point towards RE being connected to a dysregulated immune development and would also fit to the very early onset of this disease. Of note, CD4 and CD8^+^ cells were shown to express the same TCR CDR3 lengths^31^, as well as Vβ genes^32^, so a methodological bias due to an higher amount of CD4 cells in CD and TLE CNS samples as compared with RE could be excluded.

Searching for public clones in the databases revealed that the most common clone of all samples (Vβ7-9–Jβ1-1; CASSLGGTEAFF), which was found in 57% of RE patients and 65% of controls, was similar, but not identical to two published CDR3 sequences, one associated with herpes simplex immune response^33^ (CASSLGGTDTQYF) and one myelin basic protein-specific clone^34^ (CASSLGGSEETQYF), reactions against both might very well be common in both groups of patients. It has recently been shown that there are public clones of γδ T cells in the CNS of RE and CD patients^35^, and our findings support this for αβ T cells as well. As αβ T cells are much more diverse than their γδ counterparts, this is still a surprising finding. However, it remains to be seen, if any pathological or diagnostic value can be attributed to these clonotypes, if they are indeed this frequent. An RE-specific clone (found exclusively in 7 of 23 RE patients) was previously found in identical form in paediatric acquired severe aplastic anaemia^36^ (Vβ7-9–Jβ2-7; CASSPSYEQYF), which is an autoimmune disorder, where the bone marrow is attacked by the immune system. While this is only one clone, the fact that it has been found twice and both times in paediatric (putatively) autoimmune disorders might be further evidence for the relevance of CD8^+^ T cells in both disorders, but also for a perturbation of the early immune system in RE as initial trigger. Another RE-specific clone (found exclusively in 4 of 23 RE patients) was previously published to be a public clone against Epstein–Barr virus nuclear antigen 1 (Vβ7-8–Jβ2-7; CASSLGQAYEQYF)^27^. However, this is not surprising, seeing as the four RE patients in question (#01, #08, #13 and #20) were much older than the mean of the complete RE cohort (mean age of 38 years at blood draw, two of these four patients had adult-onset RE), whereas none of the controls were over the age of 25 (mean age 14.6 years). This suggests that these four RE patients have been infected with Epstein–Barr virus during the normal course of their lives, but this infection is not connected in any way to their development of RE. It does, however, validate our sequencing data set, because the appearance of such clones only in older patients shows convincingly that detection of identical clones in different patients is not due to PCR contaminations. The development of public clones has been discussed in depth, and our data support the notion of convergent recombination as an important mechanism during development of autoimmunity^37^, also with regard to nucleotide and amino-acid substitutions. Analogous to studies of different lesions in multiple sclerosis^38^, there were silent nucleotide substitutions in the TCR repertoire of RE patients, as well as controls. For example, there were 291 silent nucleotide substitutions and 21 single amino-acid substitutions in the monoclonal expansion in the CNS of patient #01. The occurrence of many substitutions is consistent with the hypothesis that these clonotypes have been selected independently during T-cell selection in the thymus and were subsequently (re)stimulated with a strong immunogenic antigen to induce a convergent, focused immune response^37^ leading to at least 291 different clones specific for the same antigen.

The finding of common HLA alleles in RE patients supports the possibility of public clones perpetuating the disease. The most prominent allele in the RE group, HLA-C*07, was phenotypically expressed in 77% of patients and accounted for 50% of all assessed HLA-C alleles. The number of patients in this study was too low to robustly assess genetic differences in a meaningful statistical way, but as a comparison the blood donor registry of Germany has assessed the allelic frequencies of 39,689 donors, and the allelic frequency of HLA-C*07 was 28% with a phenotypical expression in 50% of donors, supporting the hypothesis that antigen presentation might also be involved in the aetiology of RE. Future studies with higher numbers of patients will have to assess the relevance of this finding.

Another aspect of our study is the characterization of the TCR repertoire, and therefore T-cell plasticity, in response to treatment, as shown in individual cases: immune therapy in RE involves steroids and intravenous immunoglobulins^4^, which often do not lead to major disease modification. Patient #05 was treated with two consecutive stem cell transplantations, one autologous and one syngeneic from her identical twin sister. The TCR repertoire changed markedly after both transplantations. Monitoring TCR repertoire after the syngeneic second transplantation nicely confirms that genetically identical CD34^+^ stem cells give rise to completely different clonotypes depending on their environment^39^. This could be observed in the comparison between the different time points of patient #05, but also when comparing the TCR repertoire with that of her twin sibling. Interestingly, the putatively pathogenic clones survived/re-expanded after the autologous first immune ablation, but disappeared after the second one. As we can assume that CD34^+^ stem cells from genetically identical twins are identical as well, we propose that this might rather be due to the different regimes of chemotherapy. While the first one with cyclophosphamide and anti-thymocyte globulin apparently did not efficiently deplete all putatively pathogenic clones (potentially due to their location in the CNS), the second regime with alemtuzumab and chemotherapy following the BEAM scheme was able to sufficiently deplete all previous clonotypes allowing for complete *de novo* immune repertoire development. However, the patient did not improve after the second round of stem cell transplantation either, suggesting that the pathological/aetiological immune reaction was resumed even after the immune ablations or that maybe inflammation-independent epileptic transformation of neuronal networks and seizures had already induced lasting neurodegenerative processes. The data are consistent with the hypothesis that the antigen(s) triggering these CD8^+^ T-cell expansions might still have been present in the periphery of this patient and lead to the expansion of different, but still pathogenic clonotypes. Of note, the sequencing technique corroborated our earlier finding of a CNS-expanded clone in this patient (Vβ18-1–Jβ2-7; CASSLSGTTSYEQYF)^15^, which we now know makes up 11.6% of the CNS TCR repertoire.

Patient #08 was first treated with the anti-CD20 B-cell-depleting antibody rituximab. Thereafter, the number of unique clonotypes decreased, which is consistent with studies showing T-cell depletion after rituximab infusions^40^. However, the patient did not react favourably to the treatment. This suggests that despite small changes in the peripheral TCR repertoire after rituximab, CNS-residing pathogenic clones were not sufficiently affected. After treatment with natalizumab, the number of unique clonotypes increased markedly, consistent with its published sequestering effect^41^,^42^,^43^. Again, the putatively pathogenic clones were not depleted from the patient, but most probably restricted to the peripheral compartment. In this case, trapping the pathogenic T cells in the periphery lead to an amelioration of the disease course^20^, which is why natalizumab is today used as an experimental therapy in several RE patients.

Patient #20 was treated with basiliximab, which has been developed to help with graft-versus-host reactions/transplant rejections. However, neither did the treatment with basiliximab change the TCR repertoire nor did it completely alleviate the disease symptoms, even though the seizure frequency was reduced. The patient then suffered from a herpes zoster reactivation event and, therefore, similar to patient #08, was put on natalizumab, which had ameliorated disease symptoms in patient #08. A long-term follow-up of this patient will have to show the clinical efficacy of restricting immune cell access to the CNS. One interesting finding in patient #20 was the appearance of a very strong clonal expansion during treatment with basiliximab, which was later found in the CSF as well. An expansion of this magnitude (4.3%) in the periphery is consistent with the other findings of the strong CD8^+^ T-cell immune reaction being characteristic for RE and its disease perpetuation, but in this particular case the strong expansion might also be connected to the herpes reactivation event, even though the clone has not yet been published to be herpes-specific. The disease courses of these patients and immune repertoire response monitoring to therapy suggest that perhaps trapping T cells in the periphery might work in ameliorating RE symptoms, consistent with the hypothesis of CD8^+^ T-cell pathogenicity *in situ*.

Taken together, our study presents the TCR repertoire of RE patients in large detail, revealing pathogenically relevant information about clonotype sequences, common clones, silent nucleotide exchanges and TCR repertoire plasticity in disease, as well as during treatment.

## Methods

### RE patients and controls

In all, 23 RE patients diagnosed according to the ‘European consensus statement'^10^ were recruited in Würzburg, Bielefeld, Bonn and Münster, Germany, and included in our study. From all patients peripheral blood samples were collected and from seven of them (six brain biopsies from five patients and two CSF samples) corresponding cryopreserved diagnostic CNS biopsies were also available (Table 3). The paediatric peripheral blood control group consisted of eight patients suffering from headache, five patients suffering from a common cold, five patients suffering from TLE and five patients suffering from CD. CD patient #05 and TLE patient #05 had to be excluded from the analyses shown in Figs 1 and 3 but not from that shown in Fig. 4, as both also suffered from a common cold at the time point of blood withdrawal. The CNS controls comprised cryopreserved therapeutic resections from four CD patients and eight TLE patients, all obtained from the Department of Neuropathology, University Hospital of Bonn, Germany (Supplementary Table 1). From RE patient #01 and TLE patient #09 we had access to two brain biopsies corresponding to different brain regions. All study participants have given written informed consent. The study was approved by the local ethics committees (Ethik-Kommission der Universität Bonn (registration number 360/12), Ethik-Kommission der medizinischen Fakultät der Universität Würzburg (registration number 155/06), Ethik-Kommission der Ärztekammer Westfalen-Lippe und der Medizinischen Fakultät der Westfälischen Wilhelms-Universität Münster (registration number: 2010-245-f-S)) and was performed according to the Declaration of Helsinki.

### Peripheral blood immune cell isolation and CD8^+^ T-cell purification

Peripheral blood mononuclear cells (PBMC) were isolated by density gradient centrifugation using Lymphoprep (Progen, Heidelberg, Germany) and subsequently cryopreserved. CD8^+^ T cells were negatively isolated from thawed PBMCs using MACS magnetic cell separation (Miltenyi Biotech, Bergisch Gladbach, Germany), following the manufacturer's instructions. Purified CD8^+^ T cells were over 95% pure as assessed by staining the isolated cells with fluorescently labelled antibodies against CD3 and CD8, and subsequent flow cytometry analysis using a Gallios I flow cytometer and Kaluza 1.3 software (Beckman Coulter, Krefeld, Germany).

### RNA extraction and cDNA synthesis

Cryopreserved CNS biopsy tissue blocks were cut into 10-μm slices and directly processed with a homogenizer for optimal tissue lysis. RNA was extracted with a combination of TRIZOL (Sigma, Steinheim, Germany) and RNeasy mini columns (Qiagen, Hilden, Germany). PBMC pellets were lysed in TRIZOL, and phase separation was done using 1/10th volume of chloroform. To the aqueous phase equal volumes of 70% ethanol was added and then the manufacturer's protocol for RNeasy mini columns was followed. From the brain biopsies the RNA was extracted using only TRIZOL. cDNA of blood samples was synthesized using the Maxima Reverse Transcriptase kit with random hexamers (Thermo Scientific, Schwerte, Germany)^15^ and of CNS samples using a TCR β-chain-specific primer ‘Cβ-RT' (5′- GAA GAA GCC TGT GGC C -3′)^44^ with the Superscript III Reverse Transcriptase kit (Invitrogen, Karlsruhe, Germany).

### High-throughput T-cell receptor sequencing

CDR3β regions were amplified and sequenced by Adaptive Biotechnologies Corp (Seattle, WA) using the ImmunoSEQ assay. Briefly, a multiplex PCR system was used to amplify CDR3β sequences from cDNA samples. While the use of cDNA can lead to a biased analysis in certain cases^45^, the used sequencing depth (200,000–1,000,000 reads) in combination with single-clonal expansions consisting of thousands of reads make it improbable that the number of TCR transcripts in each of these cells making up one clonal expansion is biased in the same way. The immunoSEQ assay amplifies all 49 segments in 32 gene segment families, 8 pseudogenes segments in 7 gene families, 10 orphon segments in 10 gene families, both D genes and the 13 functional J segments. This approach generates an 87-base-pair fragment capable of identifying the VDJ region spanning each unique CDR3β (refs 17, 18). Amplicons were sequenced using the Illumina HiSeq platform. Using a baseline developed from a suite of synthetic templates, primer concentrations and computational corrections were used to correct for the primer bias common to multiplex PCR reactions^46^. Raw sequence data was filtered based on the TCRβ V, D and J gene definitions provided by the IMGT database (www.imgt.org) and binned using a modified nearest-neighbour algorithm to merging closely related sequences and remove both PCR and sequencing errors. Data were analysed using the ImmunoSEQ analyser toolset and NCBI databases. Sequences are available under the accession numbers KU748965 - KU749241 in the NCBI database (www.ncbi.nlm.nih.gov).

### Statistical analysis

Entropy was used as the read-out for TCR clonal diversity. It refers to Shannon's entropy^47^, which estimates diversity in a data set. Samples with higher entropy will have a greater diversity of sequences (=low TCR perturbation) while low-entropy samples will have many more sequences that share nucleotide identity and are thereby clonally expanded (=high TCR perturbation). Entropy is calculated by summing the frequency of each clone times the log (base 2) of the same frequency over all productive reads in a sample^48^. SO is the sum of the counts for all shared sequences observed in both samples divided by the total number of counts for all sequences (shared and unshared) in the two samples.

Data were analysed with the Student's ‘*t*-test' or linear regression including Spearman correlation using Graphpad Prism 5 software. The statistical significance is shown as *P<0.05, **P<0.005, **P<0.001 and ****P<0.0001. To detect potential disease-specific TCR Vβ expression in RE patients and Jβ gene segment usage in RE patients and controls we followed the approach used also by *Marrero et al*.^49^. The Vβ frequencies were normalized to the interval (0 or 1) by calculating (*X*_i_–*X*_min_)/(*X*_max_−*X*_min_) for each clonotype, where *X*_i_ is the frequency of an individual clonotype, and *X*_min_ and *X*_max_ represent the respective minimum and maximum. To determine the cut-off value for high- and low-frequency clonotypes within an individual patient/healthy control we calculated the *Z*-score and tested each Vβ against a two-sided significance level of 5%. Data for this part were analysed using ‘R' Version 3.1.0 (Vienna, Austria. URL http://www.R-project.org).

## Additional information

**How to cite this article:** Schneider-Hohendorf, T. *et al*. CD8^+^ T-cell pathogenicity in Rasmussen encephalitis elucidated by large-scale T-cell receptor sequencing. *Nat. Commun.* 7:11153 doi: 10.1038/ncomms11153 (2016).

## Supplementary Material

Supplementary Information Supplementary Figures 1-3 and Supplementary Tables 1-4

## Figures and Tables

**Table 1:** Sequence detail of T-cell clones shared between CNS and peripheral blood.

**Amino acid**	**TCRBV**	**TCRBJ**		
			**RE #01 Peri.**	**RE #01 CNS-i**
CASSGYEQYF	02-01	02-07	0.0652	49.1441
CASSETTPADTQYF	06-01	02-03	0.0073	4.7298
CASSSGGDRGRRAFF	07-06	01-01	0.0016	0.6821
CSASRDSLENTEAFF	20-01	01-01	0.0011	0.5186
CASSGYDYTF	02-01	01-02	0.0010	0.4086
CASTFGGELFF	27-01	02-02	0.0034	0.2431
CASSLVGVPGELFF	07-06	02-02	0.0576	0.1165
CASGIDRNWTPLHF	07-09	01-06	0.0010	0.1038
CASNTRGSTLATYNEQFF	28-01	02-01	0.0022	0.1037
CASSIGNQPQHF	19-01	01-05	0.0014	0.1036
			**RE #01 Peri.**	**RE #01 CNS-ii**
CASSGYEQYF	02-01	02-07	0.0652	44.8282
CASSGYDYTF	02-01	01-02	0.0010	0.7264
CASTFGGELFF	27-01	02-02	0.0034	0.6806
CASSETTPADTQYF	06-01	02-03	0.0073	0.6190
CSASRDSLENTEAFF	20-01	01-01	0.0011	0.2840
CASSLVGVPGELFF	07-06	02-02	0.0576	0.1568
CAISDPSPLNYGYTF	10-03	01-02	0.0899	0.1452
CASSSWTSEYTDTQYF	07-08	02-03	0.3689	0.1107
CATSRDGASTDTQYF	15-01	02-03	0.1895	0.0931
CASSSGGDRGRRAFF	07-06	01-01	0.0016	0.0896
			**RE #01 CNS-i**	**RE #01 CNS-ii**
CASSGYEQYF	02-01	02-07	49.1441	44.8282
CASSETTPADTQYF	06-01	02-03	4.7298	0.6190
CASSGEGASYNEQFF	07-09	02-01	3.2695	1.0083
CAIRGRQGAFGVWTEAFF	10-03	01-01	1.5695	5.6037
CASSDLDTGELFF	18-01	02-02	1.3142	0.2917
CASSGDILAYNEQFF	06-02	02-01	1.3133	0.0629
CASSESTGLKTQYF	06-01	02-05	0.8772	0.6730
CASSSGGDRGRRAFF	07-06	01-01	0.6821	0.0896
CSASRDSLENTEAFF	20-01	01-01	0.5186	0.2840
CASSVRPGIGYEQYF	07-02	02-07	0.4940	0.3758
			**RE #02 Peri.**	**RE #02 CNS**
CASSLGGGGDTQYF	11-02	02-03	0.0284	26.8235
CASSLAQLPATYGYTF	28-01	01-02	0.3098	1.7067
CASSLWEGGTDTQYF	28-01	02-03	0.1807	1.0436
CATHGGDDKNQPQHF	09-01	01-05	0.0047	0.3509
CASSLKLAGGRDYEQYF	28-01	02-07	0.0011	0.2337
CASSVGGFLRPQHF	02-01	01-05	0.0015	0.1434
CASSVAGGTDTQYF	09-01	02-03	0.0088	0.1406
CSVVSGGAGNEQFF	29-01	02-01	0.0135	0.1254
CASSQSGAMRDNLSGDGYTF	23-01	01-02	0.0032	0.1192
CSVAGAGAGTEAFF	29-01	01-01	0.0080	0.1117
			**RE #10 Peri.**	**RE #10 CNS**
CASSSQGDGTDTQYF	07-09	02-03	1.0298	2.3449
CASSSLTVQETQYF	07-04	02-05	0.7901	1.5062
CASSLTRGLLGELFF	14-01	02-02	4.7693	1.0646
CASSVGELAGGLDTQYF	09-01	02-03	0.4149	0.7266
CSVGTGGTNEKLFF	29-01	01-04	0.1484	0.5328
CASSPTSAAGELFF	28-01	02-02	2.8778	0.5194
CASGIGQAYEQYF	07-08	02-07	0.8002	0.3080
CASRLKGMAGELFF	13-01	02-02	1.3996	0.2588
CASSPGTGEGYEQYF	07-03	02-07	2.1107	0.2270
CSVEESRVAGVSTGELFF	29-01	02-02	0.1878	0.2215
			**RE #23 Peri.**	**RE #23 CNS**
CASRTTGPNDTQYF	07-09	02-03	0.6065	52.7764
CASSLTHPYEQYF	07-09	02-07	0.0165	8.4562
CASSIGGPEQYF	19-01	02-07	0.0219	2.6997
CASSFLRGNQETQYF	07-03	02-05	0.0212	0.7178
CASSTSGGGTDTQYF	11-02	02-03	0.0207	0.6986
CASSPSGGGTDTQYF	11-02	02-03	0.0015	0.5638
CASNFRAGPYEQYF	28-01	02-07	0.0094	0.4885
VQGICTEAFF	28-01	01-01	0.2032	0.2830
CASSPLRGGEQNQPQHF	27-01	01-05	0.0201	0.3631
CASSHRLAGTYWETQYF	07-08	02-05	0.1896	0.1493

TCRBJ, T-cell receptor joining beta chain; TCRBV, T-cell receptor variable beta chain.Listed are the shared clonotypes (amino-acid sequence, Vβ and Jβ genes, frequency in either peripheral samples (Peri.) or the CNS).

**Table 2:** Sequence detail of T-cell clones shared between CNS and peripheral blood.

**Amino acid**	**TCRBV**	**TCRBJ**	**% of peripheral RE samples**	**% of peripheral control samples**
**RE-specific common clones**				
CASSLGNQPQHF	**07-09**	01-05	52.17	0.00
CASSGYEQYF	02-01	02-07	39.13	0.00
CASSSSYEQYF	07-02	02-07	39.13	0.00
CSVEGGSSYEQYF	**29-01**	02-07	39.13	0.00
CASSDRDTGELFF	**06-04**	02-02	39.13	0.00
CASSDSNTGELFF	**06-04**	02-02	34.78	0.00
CASSPSYEQYF*	**07-09**	02-07	30.43	0.00
CASSLYNEQFF	27-01	02-01	30.43	0.00
CASSLGYEQYF	**07-09**	02-07	30.43	0.00
CSVETDTQYF	**29-01**	02-03	26.09	0.00
CASSRYEQYF	28-01	02-07	26.09	0.00
CSVGQNTEAFF	**29-01**	01-01	26.09	0.00
CSVGTGAYEQYF	**29-01**	02-07	26.09	0.00
CASSSDSYEQYF	**07-09**	02-07	26.09	0.00
CASSVGGTGELFF	**09-01**	02-02	26.09	0.00
CASSDSGTDTQYF	**06-04**	02-03	26.09	0.00
CSVGTGGTNEKLFF	**29-01**	01-04	26.09	0.00
CASSLGQGNTEAFF	05-01	01-01	26.09	0.00
CASSDSGGSYNEQFF	**06-04**	02-01	26.09	0.00
CASSDSSGSTDTQYF	**06-04**	02-03	26.09	0.00
CASSDSSGGADTQYF	**06-04**	02-03	26.09	0.00
CATSGRKRTGADTEAFF	15-01	01-01	26.09	0.00
CASSFNEQFF	28-01	02-01	21.74	0.00
CSVVEETQYF	**29-01**	02-05	21.74	0.00
CASSLGSEAFF	05-01	01-01	21.74	0.00
CASSFTGELFF	05-06	02-02	21.74	0.00
CASSYGDGYTF	06-05	01-02	21.74	0.00
CASSLGETQYF	05-06	02-05	21.74	0.00
CASSFGETQYF	**07-09**	02-05	21.74	0.00
CASSLAYEQYF	07-02	02-07	21.74	0.00
CASSLAYEQYF	05-01	02-07	21.74	0.00
CASSLLETQYF	**07-09**	02-05	21.74	0.00
CASSLGSQPQHF	**07-09**	01-05	21.74	0.00
CASSLGTDTQYF	05-01	02-03	21.74	0.00
CASSPTGSYEQYF	**07-09**	02-07	21.74	0.00
CASSIRSSYEQYF	**19-01**	02-07	21.74	0.00
CASSLGGNQPQHF	13-01	01-05	21.74	0.00
CASSLAGGNEQFF	**07-09**	02-01	21.74	0.00
CASSVAGGTDTQYF	**09-01**	02-03	21.74	0.00
CASSGTSGSTDTQYF	**06-04**	02-03	21.74	0.00
CASSDNEQFF	28-01	02-01	17.39	0.00
CAWSPNTEAFF	30-01	01-01	17.39	0.00
CASSADYGYTF	**09-01**	01-02	17.39	0.00
CAWSPGTEAFF	30-01	01-01	17.39	0.00
				
**Non-RE-specific common clones**				
CASSLGGTEAFF	**07-09**	01-01	56.52	65.22
CASSLGTGELFF	**07-09**	02-02	21.74	39.13
CASSFTDTQYF	**07-09**	02-03	26.09	30.43
CASSLAGGYEQYF	05-01	02-07	17.39	30.43
CASSIGGEQFF	**19-01**	02-01	26.09	13.04
CASSLLPSYEQYF	28-01	02-07	13.04	21.74
CASSAGDTQYF	25-01	02-03	13.04	17.39
CASSMVRGTEAFF	**19-01**	01-01	13.04	13.04
CASSLVTSGYNEQFF	**07-09**	02-01	8.70	17.39
				
**CNS-resident RE-specific common clones**				
Shared in 3 RE CNS samples				
CASSADYGYTF	**09-01**	01-02	17.39	
Shared in 2 RE CNS samples				
CASSGYEQYF	02-01	02-07	39.13	
CASSVGSNYEQYF	**09-01**	02-07	17.39	
CASSVVSTGELFF	**09-01**	02-02	17.39	
CASSETTPADTQYF	**06-01**	02-03	13.04	
CASSPYTGGVLDEQFF	**09-01**	02-01	13.04	
CASSGYDYTF	02-01	01-02	8.70	
CASSADAHTQYF	**09-01**	02-03	8.70	
CASSLTHPYDYTF	**07-09**	01-02	8.70	
CASRTTGPNDTQYF	**07-09**	02-03	8.70	
CASSESTGLKTQYF	**06-01**	02-05	8.70	
CASSFVPHSTEAFF	07-03	01-01	8.70	
CASSGEGASYNEQFF	**07-09**	02-01	8.70	
CSASRDSLENTEAFF	20-01	01-01	8.70	
CASSVGGSPTQPQHF	**09-01**	01-05	8.70	
CASSLAGGEYNEQFF	07-02	02-01	8.70	
CASSPRVGLTMSGYTF	**09-01**	01-02	8.70	
CAIRGRQGAFGVWTEAFF	10-03	01-01	8.70	
Shared in 1 RE CNS sample				
CSVGTGGTNEKLFF	**29-01**	01-04	26.09	
CASSLGQGNTEAFF	05-01	01-01	26.09	
CASSVAGGTDTQYF	**09-01**	02-03	21.74	
CASSLGPYEQYF	07-02	02-07	17.39	
CASSIDREETQYF	**19-01**	02-05	17.39	
CSGGGNEQFF	**29-01**	02-01	13.04	
CASGGNEQFF	02-01	02-01	13.04	

Listed are public clones with their amino-acid sequence, Vβ and Jβ gene, and in how many RE patients or controls this clonotype has been found. This is shown for RE-specific clones (only found in RE patients), non-RE-specific clones (found in both RE patients and controls), as well as RE-specific clones, which were also present in at least one CNS sample. The CDR3 sequence of a clone published previously in paediatric acquired severe aplastic anaemia is marked by an asterisk. The TCRBV entries highlighted in bold correspond to overexpressed genes in RE-specific clones (please see Figure 4 for details).

**Table 3:** Details of the RE patient cohort.

**#**	**Year of birth**	**HLA A**	**HLA B**	**HLA C**	**Prodromal stage**	**Acute stage**	**Age at blood draw in years**	**Early (E) or adult (A) onset**	**Hemispherectomy (age in years)**	**hemispheric ratio**
1	1994	24/30	35/50	04/06	Jun-05	Jun-05	12	E	Yes (17)	0.98
2	1999	24/74	18/57	07/07	Jul-03	Dec-04	6	E	Yes (8)	0.91
3	NA	02/02	08/56	03/07	NA	NA	NA	NA	NA	NA
4	1999	03/11	07/35	07/12	Apr-05	Apr-05	6	E	Yes	0.99
5	1988	02/02	18/50	06/07	Apr-06	Aug-06	19	A	No	NA
6	1997	01/03	04/08	07/07	May-04	May-04	8	E	No	0.84
7	2001	01/02	07/08	07/07	May-06	Jan-07	6	E	Yes (10)	0.93
8	1988	01/29	07/44	07/16	Jan-96	Jan-96	23	E	No	NA
9	1990	03/24	07/39	07/07	May-00	May-00	13	E	No	1.07
10	1955	02/11	08/51	07/15	Dec-81	Jul-06	49	A	No	0.84
11	1987	24/68	07/27	02/07	Dec-99	Aug-02	16	E	No	0.9
12	1992	24/30	13/15	01/06	Nov-01	Jan-04	12	E	No	0.97
13	1948	01/01	08/08	07/07	2003	May-04	57	A	No	0.78
14	1961	02/32	13/35:32	04/06	Nov-94	Aug-00	47	A	No	0.87
15	1993	01/02	08/39	07/12	Jun-06	Jan-09	16	E	No	0.89
16	2000	03/29	44/58	05/07	Dec-03	Aug-04	5	E	No	0.95
17	1998	24/24	35/35	04/04	Jul-04	Jul-04	7	E	No	0.88
18	2002	02/30	08/51	07/14	Oct-07	Jan-08	6	E	No	0.88
19	2001	03/32	07/18	05/07	Sep-02	Sep-03	3	E	No	0.81
20	1955	02/03	27/35	03/04	Jul-06	Jun-08	59	A	No	0.89
21	2004	02/68	15/44	03/07	Dec-08	Apr-09	11	E	No	NA
22	1980	01/02	08/18	03/07	Jan-99	Sep-10	34	A	No	0.89
23	1996	NA	NA	NA	Nov-06	Feb-07	11	E	Yes (11)	0.99

NA, not applicable.
