# Supplementary material for: CD8+ T-cell pathogenicity in Rasmussen encephalitis elucidated by large-scale T-cell receptor sequencing
Source: Nat Commun. 2016 Apr 4;7:11153. doi: 10.1038/ncomms11153 (PMC4822013; doi:10.1038/ncomms11153)
Supplement: Supplementary Information — Supplementary Figures 1-3 and Supplementary Tables 1-4 [file ncomms11153-s1.pdf]

Supplementary figure 1

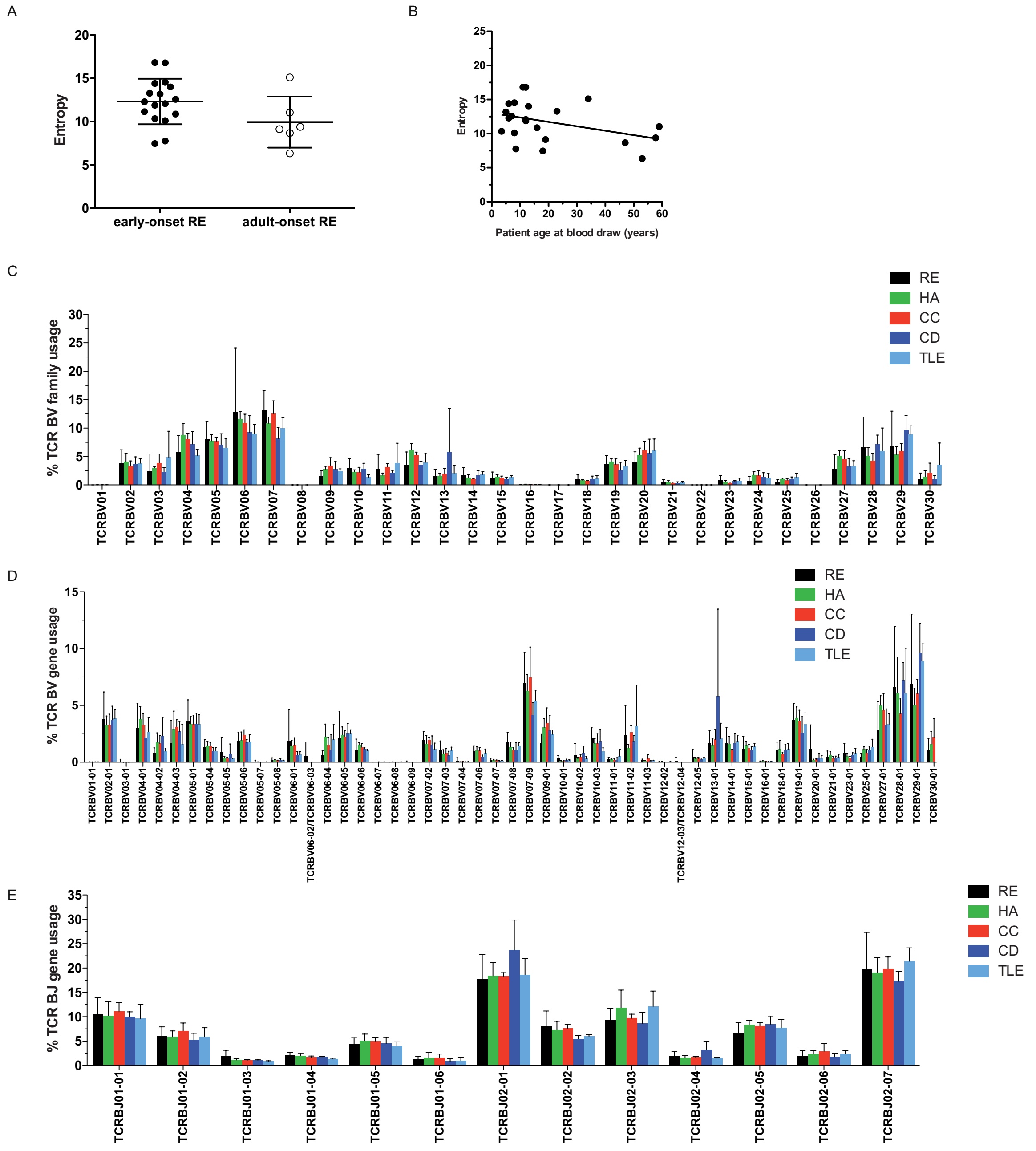

Supplementary Figure 1: Periphery
A) comparison between entropy of early-onset RE (n=17) and adult-onset RE (n=6)(not significant), B) correlation between entropy and patient age at blood draw (in years)(not significant), C) Vβ family usage in the periphery of the patient groups, D) Vβ gene usage in the periphery of the patient groups, E) Jβ gene usage in the periphery of the patient groups.

Supplementary figure 2

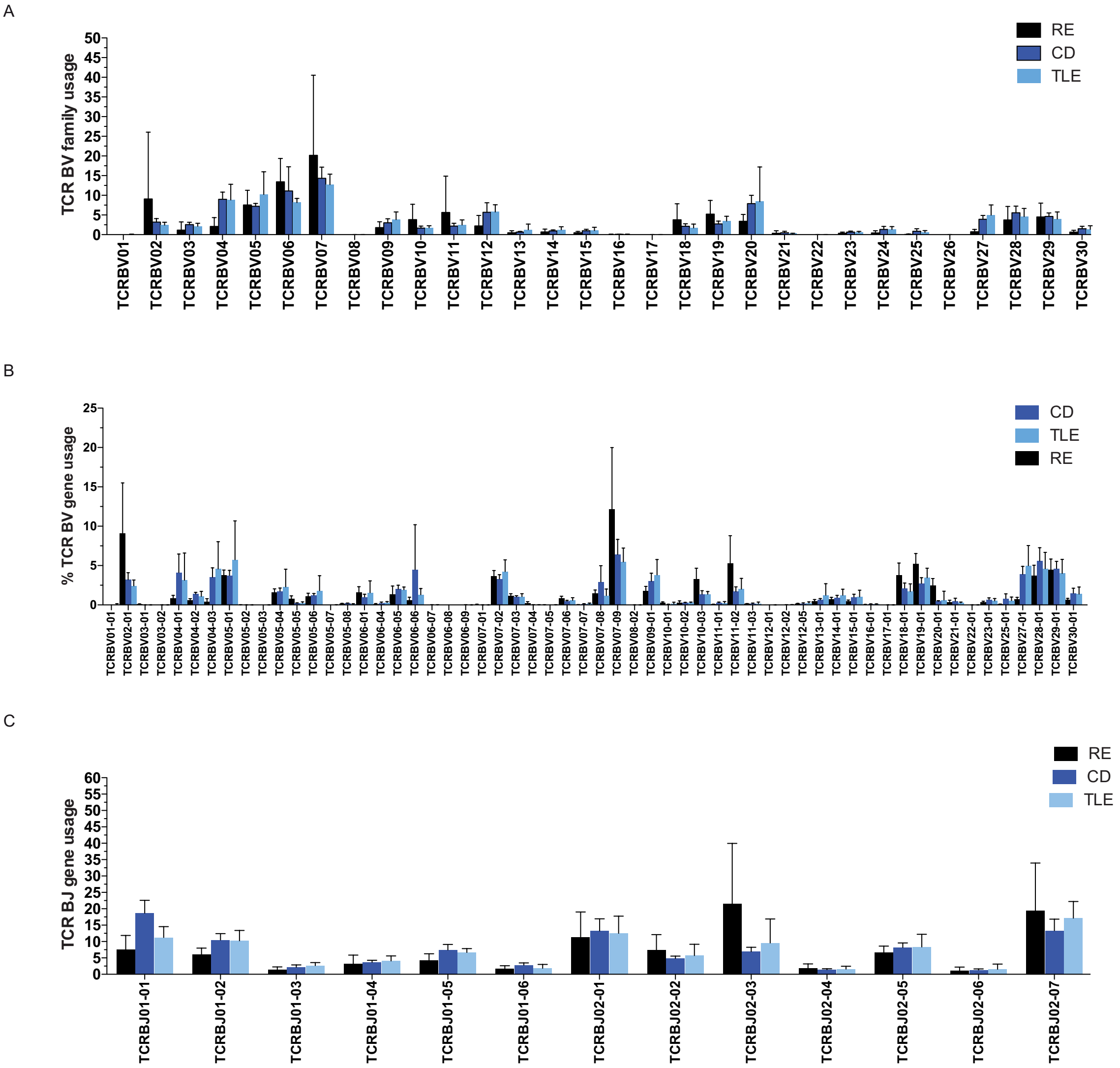

Supplementary Figure 2: CNS

A) Vβ family usage in the CNS of the patient groups, D) Vβ gene usage in the CNS of the patient groups, E) Jβ gene usage in the CNS of the patient groups.

Supplementary figure 3

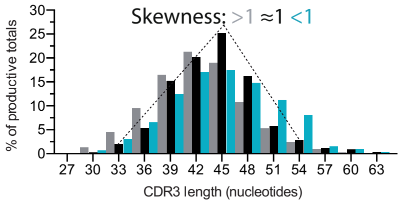

Supplementary Figure 3: Examples for skewness
Skewness is a statistical measure of the asymmetry of the probability (Gaussian) distribution of a random variable about its mean. The graph shows examples for three CDR3 length distributions: normally distributed (black, skewness=1), shifted to smaller CDR3 lengths (gray, skewness >1), or shifted to larger CDR3 lengths (blue, skewness<1).

Supplementary Table 1

#	Year of birth	Age at sampling	Diagnosis	Sample type
HA01	2001	14	HA	per. Blood CD8
HA02	2000	15	HA	per. Blood CD8
HA03	2001	14	HA	per. Blood CD8
HA04	1996	19	HA	per. Blood CD8
HA05	2000	15	HA	per. Blood CD8
HA06	2000	15	HA	per. Blood CD8
HA07	1996	19	HA	per. Blood CD8
HA08	1999	16	HA	per. Blood CD8
CC01	2005	10	CC	per. Blood CD8
CC02	2003	12	CC	per. Blood CD8
CC03	1997	18	CC	per. Blood CD8
CC04	1999	16	CC	per. Blood CD8
CC05	2006	9	CC	per. Blood CD8
CD01	1990	25	CD	per. Blood CD8
CD02	1999	16	CD	per. Blood CD8
CD03	2010	5	CD	per. Blood CD8
CD04	2000	15	CD	per. Blood CD8
CD05	2006	9	CD, CC	per. Blood CD8
CD06	n.a.	13	CD	CNS resection
CD07	n.a.	5	CD	CNS resection
CD08	n.a.	14	CD	CNS resection
CD09	n.a.	9	CD	CNS resection
TLE01	2005	10	TLE	per. Blood CD8
TLE02	2006	9	TLE	per. Blood CD8
TLE03	2001	14	TLE	per. Blood CD8
TLE04	2002	13	TLE	per. Blood CD8
TLE05	2004	11	TLE, CC	per. Blood CD8
TLE06	n.a.	11	TLE	CNS resection
TLE07	n.a.	10	TLE	CNS resection
TLE08	n.a.	14	TLE	CNS resection
TLE09	n.a.	13	TLE	CNS resection (2x)
TLE10	n.a.	5	TLE	CNS resection
TLE11	n.a.	11	TLE	CNS resection
TLE12	n.a.	10	TLE	CNS resection
TLE13	n.a.	44	TLE	CNS resection

Details of the control patient cohort

Supplementary Table 2

Patient	RE #01	RE #02	RE #23	TLE #11	CD #05
**Silent nucleotide substitutions**	291	11	18	20	5
**Single amino acid substitutions**	21	1	22	5	2
**Amino acid sequence**	CASSGYEQYF	CASSLGGGGDTQYF	CASRTTGPNDTQYF	CASSSGRTGTDYEQYF	CASSPATEAFF
**CDR3 length (nucleotides)**	30	42	42	48	33
**TCRVB**	TCRBV02-01	TCRBV11-02	TCRBV07-09	TCRBV05-01	TCRBV06-06
**TCRJB**	TCRBJ02-07	TCRBJ02-03	TCRBJ02-03	TCRBJ02-07	TCRBJ01-01
**Frequency (CNS)**	49.1%	26.8%	52.8%	16.0%	12.6%
**Substituted clones**	CATSGYEQYF	GASSLGGGGDTQYF	CASRTAGPNDTQYF	CASSPGRTGTDYEQYF	CARSPATEAFF
	CAISGYEQYF		CASRTPGPNDTQYF	CASSQGRTGTDYEQYF	CASSTATEAFF
	CGSSGYEQYF		CASRTTGPSDTQYF	CASSSGRTGTDYEPYF	
	CASSGSEQYF		CASRTTGPRDTQYF	CASSSGRTGTDYELYF	
	CASRGHEQYF		CASRTTGPTDTQYF	CASSSGRPGTDYEQYF	
	CASSGDEQYF		CASRPTGPNDTQYF		
	GASSGYEQYF		CASRATGPNDTQYF		
	FASSGYEQYF		RASRTTGPNDTQYF		
	CASSGGEQYF		CGSRTTGPNDTQYF		
	WASSGYEQYF		WASRTTGPNDTQYF		
	CASSTYEQYF		GASRTTGPNDTQYF		
	CASSEYEQYF		CASRTTGPNETQYF		
	CASSRYEQYF		CASRTTGPNGTQYF		
	CASSQYEQYF		CASRTTGPNDAQYF		
	CASSKYEQYF		CASSTTGPNDTQYF		
	CASSDYEQYF		CPSRTTGPNDTQYF		
	CASSPYEQYF		CCSRTTGPNDTQYF		
	CASSWYEQYF		CTSRTTGPNDTQYF		
	CASSSYEQYF		FASRTTGPNDTQYF		
	CPSSGYEQYF		SASRTTGPNDTQYF		
	CASSGCEQYF		CARRTTGPNDTQYF		
			CASSTTGPNDTQYF		

Listed are the number of silent nucleotide substitutions, single amino acid substitutions, amino acid sequence of the expansion, CDR3 length in nucleotides, Vβ and Jβ genes, frequency in the respective CNS, and the clonotypes with the single amino acid substitutions (with the substitution in red).

Supplementary Table 3

Ctrl-specific common clones				
amino acid	TCRBV	TCRBJ	% of peripheral RE samples	% of peripheral control samples
CASSQEGGISEAFF	TCRBV04-03	TCRBJ01-01	0	73.91
CASSQEKGDQPQHF	TCRBV04-01	TCRBJ01-05	0	65.22
CASSSSAGGPTYEQYF	TCRBV27-01	TCRBJ02-07	0	60.87
CASSETGGSYNEQFF	TCRBV04-01	TCRBJ02-01	0	39.13
CASSPTSFGDNEQFF	TCRBV05-06	TCRBJ02-01	0	39.13
CSVVNNEQFF	TCRBV29-01	TCRBJ02-01	0	34.78
CAWSGRGEQFF	TCRBV30-01	TCRBJ02-01	0	34.78
CASRQGWNEQFF	TCRBV19-01	TCRBJ02-01	0	34.78
CASSPVQDNEKLFF	TCRBV18-01	TCRBJ01-04	0	34.78
CASSFLDSDQPQHF	TCRBV28-01	TCRBJ01-05	0	30.43
CASSDVGYGNTIYF	TCRBV25-01	TCRBJ01-03	0	30.43
CATSRDRGNEKLFF	TCRBV15-01	TCRBJ01-04	0	30.43
CASSPLDGLRNEQFF	TCRBV28-01	TCRBJ02-01	0	30.43
CASRERGQSTGELFF	TCRBV19-01	TCRBJ02-02	0	30.43
CASSMSGARAKNIQYF	TCRBV28-01	TCRBJ02-04	0	30.43
CASSDPAEQTTDTQYF	TCRBV02-01	TCRBJ02-03	0	30.43
CASSLRPDTQYF	TCRBV13-01	TCRBJ02-03	0	26.09
CALDISGANVLTF	TCRBV30-01	TCRBJ02-06	0	26.09
CSVRGGAPQETQYF	TCRBV29-01	TCRBJ02-05	0	26.09
CASSWSGTHNEQFF	TCRBV27-01	TCRBJ02-01	0	26.09
CASTYSGGDTEAFF	TCRBV09-01	TCRBJ01-01	0	26.09
CASSVKGGTDTQYF	TCRBV06-04	TCRBJ02-03	0	26.09
CASSAPGLVAGPQYF	TCRBV09-01	TCRBJ02-05	0	26.09
CASSEVAGVGDTQYF	TCRBV10-02	TCRBJ02-03	0	26.09
CASSLRWVSLNTEAFF	TCRBV27-01	TCRBJ01-01	0	26.09
CASSPPSGGAQETQYF	TCRBV04-01	TCRBJ02-05	0	26.09
CATSRGTSDSTDTQYF	TCRBV15-01	TCRBJ02-03	0	26.09
CASSDESETQYF	TCRBV18-01	TCRBJ02-05	0	21.74
CASSRDRAQQYF	TCRBV14-01	TCRBJ02-07	0	21.74
CASSLLYMDTQYF	TCRBV19-01	TCRBJ02-03	0	21.74
CASTRQVYQETQYF	TCRBV28-01	TCRBJ02-05	0	21.74
CASSLRAPTGELFF	TCRBV28-01	TCRBJ02-02	0	21.74
CASSFGGSSYEQYF	TCRBV14-01	TCRBJ02-07	0	21.74
CASTHWRGTDTQYF	TCRBV23-01	TCRBJ02-03	0	21.74
CASSLNYVEKKLFF	TCRBV05-06	TCRBJ01-04	0	21.74
CASSSPGQANEKLFF	TCRBV05-01	TCRBJ01-04	0	21.74
CASSLSDLGRTDEQYF	TCRBV27-01	TCRBJ02-07	0	21.74
CATPPGTSGNTDTQYF	TCRBV23-01	TCRBJ02-03	0	21.74
CASSSSSGVSSYNEQFF	TCRBV09-01	TCRBJ02-01	0	21.74
CASSLGSSREGPDTQYF	TCRBV13-01	TCRBJ02-03	0	21.74
CASSPRTDGYTF	TCRBV07-02	TCRBJ01-02	0	17.39
CASRDTGTEAFF	TCRBV06-05	TCRBJ01-01	0	17.39
CAWAASPDSEQYF	TCRBV30-01	TCRBJ02-07	0	17.39
CATSREGREYGYTF	TCRBV15-01	TCRBJ01-02	0	17.39
CASNQQGVNTEAFF	TCRBV02-01	TCRBJ01-01	0	17.39
CASSLAAGVETQYF	TCRBV05-06	TCRBJ02-05	0	17.39
CAWSVQSGTYEQYF	TCRBV30-01	TCRBJ02-07	0	17.39
CASTDSGSLYEQYF	TCRBV05-01	TCRBJ02-07	0	17.39
CASSPTGRGEKLFF	TCRBV25-01	TCRBJ01-04	0	17.39
CASRDRGSTNEKLFF	TCRBV04-03	TCRBJ01-04	0	17.39

Listed are the clonotypes (amino acid sequence, Vβ and Jβ genes, frequency in either RE patients or control patients). Vβ genes in green are over-expressed in RE-specific clones (please see Figure 4 for details and Table 2 for the respective list of RE-specific clones).

Supplementary Table 4

Amino acid	TCRBV	TCRBJ				
			**RE #05-a**	**RE #05-CNS**		
CASSDYAGVTDTQYF	06-01	02-03	0.1265	0.3426		
CASSKGQPSEQFF	19-01	02	0.1960	0.2840		
CASSVLGRGASEQFF	09-01	02-01	0.0086	0.0538		
CASSLGQGYEQYF	27-01	02-07	0.0051	0.0227		
CASSKGQPSDYTF	19-01	01-02	0.0002	0.0052		
			**RE #05-b**	**RE #05-CNS**		
CATPGPTGELFF	10-03	02-02	0.1171	8.1123		
CSGAQGGEKLFF	29-01	01-04	0.0092	3.2679		
CASSMRSTDTQYF	19-01	02-03	0.9580	1.7971		
CASSYNYGYTF	07-02	01-02	0.0053	1.7530		
CASSLEAGSNEKLFF	07-06	01-04	0.0437	0.4094		
CASSDYAGVTDTQYF	06-01	02-03	1.4232	0.3426		
CASSKGQPSEQFF	19-01	02	0.0960	0.2840		
CASSDPSSYNSPLHF	02-01	01-06	0.0259	0.1200		
CASSRTGENQPQHF	27-01	01-05	0.0016	0.0830		
CASSLDGPLDEQYF	11-02	02-07	0.0066	0.0804		
			**RE #05-a**	**RE #05-b**		
CASSDYAGVTDTQYF	06-01	02-03	0.1265	1.4232		
CASSLRGTEAFF	05-06	01-01	0.2911	0.5471		
CASSFPLTGRMDTEAFF	07-09	01-01	0.1560	0.2429		
CSVEDPDVHGGYTF	29-01	01-02	0.1465	0.2185		
CASSKGQPSEQFF	19-01	02	0.1966	0.0965		
CASSQEAGVYYNEQFF	04-01	02-01	0.0875	0.0619		
CASSPGAGITEAFF	07-09	01-01	0.0082	0.1425		
CASSEARGLAVQETQYF	02-01	02-05	0.0926	0.0484		
CSVEVNTEAFF	29-01	01-01	0.0668	0.0625		
CSARKEGVYNEQFF	20-01	02-01	0.0575	0.0681		
			**RE #05-a**	**RE #05-b**	**RE #05-CNS**	
CASSDYAGVTDTQYF	06-01	02-03	0.1265	1.4232	0.3426	
CASSKGQPSEQFF	19-01	02	0.1966	0.0965	0.2840	
			**RE #05-a**	**ST**		
CASSVQGNTEAFF	19-01	01-01	0.0175	0.0007		
CASSLGGGYEQYF	27-01	02-07	0.0005	0.0007		
			**RE #05-b**	**ST**		
CASSLGKNIQYF	11-02	02-04	0.0247	5.8354		
CASSLGNEQFF	07-08	02-01	0.0010	2.4605		
CSVGTGGTNEKLFF	29-01	01-04	0.0574	0.0049		
CASSFSYEQYF	13-01	02-07	0.0368	0.0007		
CASSLRETQYF	07-09	02-05	0.0260	0.0007		
CASSLGRGTEAFF	05-01	01-01	0.0232	0.0007		
CASSLGGDQPQHF	11-02	01-05	0.0164	0.0021		
CASSDTGELFF	07-06	02-02	0.0147	0.0010		
CASSLGEKLFF	07-09	01-04	0.0101	0.0007		
CASSEDGMNTEAFF	10-02	01-01	0.0086	0.0049		
			**RE #05-CNS**			
CASSLSGTTSYEQYF	18-01	02-07	11.6287			
CATPGPTGELFF	10-03	02-02	8.1123			
CASSPPRTGELFF	06	02-02	3.8914			
CSGAQGGEKLFF	29-01	01-04	3.2679			
CSVEDPLGGGYSNQPQHF	29-01	01-05	2.9435			
CSVGAQLAPTHTDTQYF	29-01	02-03	2.9273			
CASSLEATVRGEKLFF	05-01	01-04	2.1591			
CASSLTQGRTDTQYF	05-04	02-03	2.1500			
CAISSGGLRETQYF	10-03	02-05	1.9424			
CASSETGTSNTGELFF	07-09	02-02	1.8620			
			**RE #08-a**	**RE #08-CSF**		
CASSITDTQYF	09-01	02-03	0.0009	1.7383		
CASSPPSGSKGEQFF	18-01	02-01	0.0969	1.4450		
CASRMTSGSSGELFF	14-01	02-02	0.0008	1.3351		
CASSPTASTNEKLFF	06-05	01-04	0.0826	0.9873		
CASSLVTGNGEKLFF	07-06	01-04	0.0833	0.9827		
CASSALAGPYEQYF	28-01	02-07	0.0107	0.9334		
CASSSLGQGAKNIQYF	12	02-04	0.0883	0.6934		
CASSQKERGGSSTDTQYF	03	02-03	0.0852	0.5596		
CASSRLGQETQYF	07-06	02-05	0.0049	0.5076		
CASSQTNAGGEQYF	03	02-07	0.0020	0.4488		
			**RE #08-B**	**RE #08-CSF**		

CASSITDTQYF	09-01	02-03	0.0010	1.7384		
CASSPPSGSKGEQFF	18-01	02-01	0.0030	1.4451		
CASSPTASTNEKLFF	06-05	01-04	0.0288	0.9874		
CASSLVTGNGEKLFF	07-06	01-04	0.0234	0.9828		
CASSYVGQQNTEAFF	06-05	01-01	0.0023	0.9372		
CASSALAGPYEQYF	28-01	02-07	0.0075	0.9335		
CASSSLGQGAKNIQYF	12	02-04	0.0681	0.6935		
CASSQKERGGSSTDTQYF	03	02-03	0.0020	0.5597		
CASSEVSAGTGVYEQYF	02-01	02-07	0.0287	0.5364		
CASSLERTDTQYF	05-01	02-03	0.0026	0.5085		
			**RE #08-c**	**RE #08-CSF**		
CASSITDTQYF	09-01	02-03	0.0020	1.7384		
CASSPPSGSKGEQFF	18-01	02-01	0.0017	1.4451		
CASSPTASTNEKLFF	06-05	01-04	0.0422	0.9874		
CASSLVTGNGEKLFF	07-06	01-04	0.0555	0.9828		
CASSALAGPYEQYF	28-01	02-07	0.0094	0.9335		
CASSSLGQGAKNIQYF	12	02-04	0.1113	0.6935		
CASSQKERGGSSTDTQYF	03	02-03	0.0004	0.5597		
CASSEVSAGTGVYEQYF	02-01	02-07	0.0150	0.5364		
CSVEGQIQETQYF	29-01	02-05	0.0007	0.4393		
CASRLNTEAFF	06-05	01-01	0.0194	0.4387		
			**RE #08-a**	**RE #08-b**	**RE #08-c**	**RE #08-CSF**
CASSITDTQYF	09-01	02-03	0.0009	0.0010	0.0020	1.7384
CASSPPSGSKGEQFF	18-01	02-01	0.0969	0.0030	0.0017	1.4451
CASSPTASTNEKLFF	06-05	01-04	0.0827	0.0288	0.0422	0.9874
CASSLVTGNGEKLFF	07-06	01-04	0.0833	0.0234	0.0555	0.9828
CASSALAGPYEQYF	28-01	02-07	0.0107	0.0075	0.0094	0.9335
CASSSLGQGAKNIQYF	12	02-04	0.0883	0.0681	0.1113	0.6935
CASSQKERGGSSTDTQYF	03	02-03	0.0853	0.0020	0.0004	0.5597
CASRLNTEAFF	06-05	01-01	0.0221	0.0100	0.0194	0.4387
CASSSLNQPQHF	05-06	01-05	0.0073	0.0041	0.0178	0.4152
CASSLVGIVDEQYF	05-04	02-07	0.0088	0.0039	0.0008	0.4121
			**RE #08-CSF**			
CASSITDTQYF	28-01	02-03	1.7384			
CASLVNTEAFF	06-06	01-01	1.5032			
CASSPPSGSKGEQFF	18-01	02-01	1.4451			
CASSFLGTGANVLTF	07-09	02-06	1.3685			
CASRMTSGSSGELFF	14-01	02-02	1.3352			
CASSYGENEKLFF	06-05	01-04	1.1276			
CASSSGTSLNEQFF	06-05	02-01	1.0624			
CASSPTASTNEKLFF	06-05	01-04	0.9874			
CASSLEGVNTQYF	11-02	02-03	0.9834			
CASSLVTGNGEKLFF	07-06	01-04	0.9828			
			**RE #20-a**	**RE #20-CSF**		
CASSQGPTQYF	04-03	02-05	0.0032	0.7655		
CASNYITSGYEQYF	07	02-07	0.5246	0.7508		
CATRIGDTEAFF	19-01	01-01	0.0027	0.7030		
CATDTGPDSYEQYF	10-03	02-07	0.9471	0.6136		
CASSPHRGPSDTQYF	07-08	02-03	0.1082	0.5695		
CASSTDSETLTGELFF	12	02-02	0.3818	0.5548		
CAIRSMNTEAFF	10-03	01-01	0.0015	0.4581		
CASDTRDSNQPQHF	19-01	01-05	0.3212	0.4005		
CASSGGGSFSYEQYF	19-01	02-07	0.0101	0.3135		
CASSMGTGDHEQYF	19-01	02-07	0.0998	0.3050		
			**RE #20-b**	**RE #20-CSF**		
CASNYITSGYEQYF	07	02-07	0.2133	0.7508		
CASSIEGRSTQPQHF	19-01	01-05	0.0010	0.6197		
CATDTGPDSYEQYF	10-03	02-07	0.3448	0.6136		
CASSPHRGPSDTQYF	07-08	02-03	0.0399	0.5695		
CASSTDSETLTGELFF	12	02-02	0.0972	0.5548		
CASDTRDSNQPQHF	19-01	01-05	0.2390	0.4005		
CATLASGQETQYF*	07-09	02-05	4.3104	0.3540		
CASSGGGSFSYEQYF	19-01	02-07	0.0045	0.3135		
CASSMGTGDHEQYF	19-01	02-07	0.1077	0.3050		
CASSSRASGGQETQYF	28-01	02-05	0.2021	0.2976		
			**RE #20-a**	**RE #20-b**	**RE #20-CSF**	
CASNYITSGYEQYF	07	02-07	0.5246	0.2133	0.7508	
CATDTGPDSYEQYF	10-03	02-07	0.9471	0.3448	0.6136	
CASSPHRGPSDTQYF	07-08	02-03	0.1082	0.0399	0.5695	
CASSTDSETLTGELFF	12	02-02	0.3818	0.0972	0.5548	
CASDTRDSNQPQHF	19-01	01-05	0.3212	0.2390	0.4005	
CASSGGGSFSYEQYF	19-01	02-07	0.0101	0.0045	0.3135	

CASSMGTGDHEQYF	19-01	02-07	0.0998	0.1077	0.3050	
CASSSRASGGQETQYF	28-01	02-05	0.2782	0.2021	0.2976	
CASSSEPGLAVNTQYF	05-01	02-03	0.0016	0.0020	0.2621	
CASSPGPGEKLFF	12	01-04	0.0267	0.0040	0.2486	
			**RE #20-CSF**			
CASSTQSYKAFF	07-02	01-01	1.0178			
CASSQGPTQYF	04-03	02-05	0.7545			
CASNYITSGYEQYF	07	02-07	0.7459			
CATRIGDTEAFF	19-01	01-01	0.7030			
CASSFQGLDGYTF	07-02	01-02	0.6810			
CATDTGPDSYEQYF	10-03	02-07	0.6136			
CASSLDIDGYTF	07-02	01-02	0.5903			
CASSFTRRQQETQYF	07-09	02-05	0.5732			
CASSTDSETLTGELFF	12	02-02	0.5548			
CASSIEGRSTQPQHF	19-01	01-05	0.5438			

Listed are the shared clonotypes (amino-acid sequence, Vb and Jb genes, frequency in either peripheral samples (peri.) or the CSF). The CDR3 sequence marked by an asterisk expanded during treatment with basiliximab to the highest peripheral frequency (4.3%) and could later be found in the CSF.
